# Veterinary Medical Register

**Published:** 1881-07

**Authors:** 


### VETERINARY MEDICAL REGISTER

—OF THE—

### UNITED STATES.

COMPILED FOR THE

### *JOURNAL OF COMPARATIVE MEDICINE.*

[COPYRIGHTED: ALL RIGHTS RESERVED.]

The accompanying Register has been compiled with much labor and care. It is believed to include a very large majority of the veterinary practitioners of this country. A supplement, however, will be issued in a subsequent number of the JOURNAL OF COMPARATIVE MEDICINE which will include all names sent to or secured by the editor during the ensuing three months.

We are indebted to a large number of medical, agricultural, and sporting journals for inserting our request that veterinarians send us their addresses. We are also indebted to Mr. W. H. Clark and to Mr. W. R. Jenkins, publisher, for supplying us with lists of names.

It will be seen that but few of the names have any titles appended. We have purposely put on the title D. V. S. only when there was evidence, from college catalogues or the written statements of veterinarians, that such title was due them. We have also only appended the title V. S. in cases where such title has been distinctly assumed in correspondence with us or others. There remains a very large number of persons who are put down in directories and elsewhere as veterinary surgeons, but whose exact title we do not know. We have, therefore, given them no title at all. All corrections in this matter of title we shall be very glad to receive, and they will he attended to in a subsequent edition of the Register.

In connection with the publication of this Register, we wish to call attention to a few striking facts regarding veterinary medicine in this country. There are in the present Register 898 names. We do not think that the total number of regular veterinary surgeons in this country greatly exceeds this. Of these ——— there are, as nearly as we can estimate, not over 200 veterinarians who have received a regular professional education. The rest have picked up what knowledge they could in various ways, and have then assumed the title. Their education and professional skill is necessarily far below what could be desired.

Now, on the other hand, there were in this country in 1878 the following number of domestic animals:

| | |
|---|---|
| Horses, - - - - - - - | 10,938,700 |
| Mules and asses, - - - - - - | 1,713,100 |
| Cattle, - - - - - - - | 33,234,500 |
| Sheep, - - - - - - - | 38,123,800 |
| Swine, - - - - - - - | 34,766,100 |
| Total, - - - - - - | 118,776,200 |

A very low estimate of the value of the above is $2,000,000,000. This does not include dogs, hens, and other domestic animals. The annual loss among all these creatures from various diseases and injuries is simply immense.

From one disease alone, hog cholera, there has been an annual loss of, it is estimated, $200,000,000. Devastating epidemics are likely to break out any year.

The loss to exporters on account of pleuro-pneumonia was in one year about $2,250,000.

We place these vast moneyed interests on one hand, and the very small number of educated veterinarians, who form an important element in protecting them, on the other. The necessity for more skilled practitioners is apparent, and is, indeed, a crying one.

It is estimated that there are nearly twice as many physicians in this country as are really needed.

There are, on the other hand, probably not one-twelfth as many fairly good veterinarians as our commercial and agricultural interests require or as our sympathies with brute suffering should demand.

Addams, J. W., Cedarville, Ill.
Agenburg, G. S., Vermillion, Dakota.
Alba, P. F., Mobile, Ala.
Alexander, J., V.S., Manchester, N.H
Alger, D., Providence, R. I.
Allen, E. R., Toledo, Kansas.
Allen, G. W., V.S., Frankford, (Philadelphia) Pa.
Alloway, C. J., D.V.S., Montreal, Canada.
Andinwood, S. T., Albany, N. Y.
Arminger, J., Baltimore, Md.
Atchison, J., New York, N. Y.
Atherton, T. W., Rockland, Me.
Atkins, C. H., Chicago, Ill.
Atkinson, V. T., Milwaukee, Wis.
Audrain, H., D.V.S., Montreal, Can.
Aubenreith, J., Hudson City, N. Y.
Ayer, J. B., Omaha, Neb.
Bailey, ——, New York, N. Y.
Bailey, G. H., D.V.S., Portland, Me.
Baker, A. H., V.S., Chicago, Ill.
Baker, J. D., Winafall, Ind.
Baker, J. J., West Troy, N. Y.
Baker, M. C., D.V.S., Montreal, Canada.
Baker, W., Brooklyn, N. Y.
Baker, W., Washington, D. C.
Ball, E. P., Derby Line, Vt.
Bardock, A. R., Yale, Kansas.
Barker, G. D., Syracuse, N. Y.
Barker, M. O. Rochester, N. Y.
Barling, E., Utica, N. Y.
Barron, T. F. & Son, Baltimore, Md.

Bartlett, J. G., Chicago, Ill.
Bartram, E. W., Ann Arbor, Mich.
Bassett, G, W., Dayton, O.
Bates, E. S., M.D.V.S., New York, N. Y.
Bausenberger, H., Minneapolis, Minn.
Bavett, J., Fort Omaha, Neb.
Bayes, R. B., Milwaukee, Wis.
Beal, F. E. S., Ames, Iowa.
Becker, ——, Brooklyn, N. Y.
Beech, — —, New York, N. Y.
Bell, G. W., Oswego, N. Y.
Bell, J., Montreal, Canada.
Bell, L. T., V.S., Brooklyn, N. Y
Bell, S. J., Memphis, Tenn.
Bell, S. J., D. V. S., San Francisco, Cal.
Bell, W., Kars, Ontario.
Benedict, J. N., V.S., Albion, Mich.
Bennett, D. A., Cleveland, O.
Bennett, P., Brooklyn, N. Y.
Bentley, H. A., Mountain City, Nev.
Bergevin, H., D.V.S., St. Timothée, P. Q.
Berkman, C. C. & Son, St. Paul,Minn.
Berkman, D. M., Rochester, Minn.
Berns, G. S., D.V.S., Brooklyn, N. Y.
Berry, J., V.S., Marion, O.
Berry, J., Philadelphia, Pa.
Berry, J. B., Philadelphia, Pa.
Bessailon, H., V.S., St. Valentine,P.Q
Bessey, C. E., Ph.D., Ames, Iowa.
Billings, F. S., V.G., Boston, Mass.
Birch, J., Pittsburg, Pa.
Birch, M. W., Philadelphia, Pa.
Bissell, G. B., Springfield, Mass.
Blackwood, T., D.V.S., Boston, Mass.
Blake, S., Ipswich, Mass.
Blakely, R. P., D.V.S., Syracuse, N. Y.
Block, H., Cincinnati, O.
Blodgett, A. Z., Waukegan, Ill.
Blumenthal, A., Hartford, Ct.
Bodelock, A., Grand Rapids, Mich.
Bohman, W., Chicago, Ill.
Bond, J. P., Toronto, Can.
Bonis, P. D., San Francisco, Cal.
Berz, J., Syracuse, N. Y.
Bossert, W. H., Oaks, Pa.
Boswell, C., Chicago, Ill.
Bovett, J. A., Chicago, Ill.
Bowen, E. S., Chicago, Ill.
Bowler, G. W., M.D., V.S., Cincinnati, O.
Bowman, E. G., Pittsburg, Pa.
Bowman, E. P., Lynn, Mass.
Bowyer, G. A., Jr., Pine Grove, Ky.
Boyd, H. B., D.V.S., New Rochelle, N. Y.
Boyle, C. C., New Orleans, La.
Brackett, E. J., Minneapolis, Minn.
Brackett, G. H., Worcester, Mass.
Bray, P. A., Chicago, Ill.
Brotherton, W. C., New York, N. Y.
Brewster, B. S., Norwich, Ct.
Bridge, F., Philadelphia, Pa.
Briggs, D., Pawtucket, R. I.,
Bright, W., Chelsea, Mass.
Brown, D. S., D.V.S., Roscoe, Ill.
Brown, G. E. & Co., Aurora, Ill.
Brown, G. W., Pittsburg, Pa.
Brown, J. S., Dayton, O.
Brown, M. S., D.V.S., Montreal, Can.
Bruneau, O., D.V.S., Montreal, Can.
Bryden, W., Boston, Mass.
Buchanan, A. C., Providence, R. I.
Bucher, C., Milwaukee, Wis.
Buckalew, W. H., Rochester, N. Y.
Budd, —, New York, N, Y.
Bullinoer, S., Philadelphia, Pa.
Bunker, M., D.V.S., New York, N.Y.
Bunsbury, F. A., V.S., Niles, Mich.
Burden, C., V.S., New York, N. Y.
Burdett, S., V.S., Billerton, Kans.
Burke, T. J., New York, N. Y.
Burns, P., San Francisco, Cal.
Burton, J. S., Hartford, Ct.
Bushman, J., Washington, D. C.
Buzzard, A., Syracuse, N. Y.
Byers, G. W., Sycamore, O.
Byrne, J. M., Bangor, Me.
Caldwell, H., Dunbarton, N. H.
Callahan, D. J., Chicago, Ill.
Callum, C., Meadville, Pa.
Canfield, W. J., Atchison, Kans.
Carmody, W. T., V.S., New York, N. Y.
Carpenter, W. H., D.V.S., San Francisco, Cal.
Carroll, J. H., Rochester, N. Y.
Carter, E. J., Montreal, Can.
Casewell, J., D.V.S., San Francisco, Cal.
Castle, T. H., Wilmington, Del.
Cattanach, C. C., D.V.S., New York, N. Y.
Cattanach, J. S., New York, N. Y.
Cawley, W. W., Selinsgrove, Pa.
Chadwick, E. B., Brookline, Mass.
Chadwick, W., Chicago, Ill.

Chamberlain, W. I., Buffalo, N. Y.
Chambers, S. B., Toledo, O.
Chanbon, E., Jersey City, N. J.
Chandler, J., Montreal, Can.
Charlesworth, J., Springfield, O.
Chase, J. S., Jr., Fall River, Mass.
Chase, L. P., Ashland, O.
Chevalier, J. B., Montreal, Can.
Chittenden, E.H., Minneapolis, Minn.
Churchman & Jackson, Indianapolis, Ind.
Clark, J., Kempton, Ill.
Clark, L. J., Chicago, Ill.
Claussen, —, San Francisco, Cal.
Clemens, C. S., Fremont, Neb.
Clever, W. E., Washington, D. C.
Coates, W. J., D.V.S., New York, N. Y.
Cochran, D. W., D.V.S., New York, N. Y.
Colburn, C., Boson, Mass.
Colburn, C., West Dedham, Mass.
Coleman, D., Wolfboro, N. H.
Collins, W. H., Cincinnati, O.
Colsson, P. Z., D.V.S., Mobile, Ala.
Conedy, F. Z., Shelbourne Falls, Mass.
Cooley, S. W. P., Chicago, Ill.
Corbyn, T. N., Philadelphia, Pa.
Corlies, J. C., Newark, N. J.
Cormack, A. C., Chicago, Ill.
Cosgrove, J. B., V.S., Worcester, Mass.
Casson, P. Z., Mobile, Ala.
Cotton, T. B., V.S., Mount Vernon, O.
Courtenay, P. R., Baltimore, Md.
Couture, J. A., D.V.S., Quebec, Can.
Cowan, J., Memphis, Tenn.
Cowhey, T. C.,D.V.S., St. Louis,Mo.
Cowman, W. M., Cincinnati, O.
Crabb, C., Cleveland, O.
Craig, W. M., Chicago, Ill.
Crane, H., V.S., Pike, N. Y.
Crane, L., M.D.,V.S., Paterson, N. J.
Crapo, H., Bridgewater, Mass.
Cressy, Noah, M.D., V.S., Ph.D., Hartford, Ct.
Crohn, M., Chicago, Ill.
Crombie, N., Peterboro, N. H.
Crowley,C.W., D.V.S.,St. Louis,Mo.
Croxon, R. & Son, Detroit, Mich.
Crutton, C. H., Providence, R. I.
Cuff, J., D.V.S., New York, N. Y.
Cummins, P., D.V.S., Quebec, Can.
Cunnes, J., Philadelphia, Pa.
Cupps, A. J., Pittsburg, Pa.
Curran, J., Brooklyn, N. Y.
Cushman, J., Chicago, Ill.
Cutler, F., New York, N. Y.
Cutting, A. M., South Braintree, Mass.
Cutting, W., Rochester, N. Y.
Czarwecki, E., Pittsburg, Pa.
Dabazies, A., New Orleans, La.
Dailey, J. K., Chicago, Ill.
Dance, J. W., Philadelphia, Pa.
Dancer, J. H., D.V.S., New York, N. Y.
Darling,S.A.,D.V.S.,New York,N.Y.
Daubigny, V. T., D.V.S., Lachenaie, P. Q.
Davis, C., Peabody, Mass.
Day, S., Charlestown, Mass.
Dechard, J. B., Middletown, Pa.
De Graff, C. A., Janesville, Minn.
De Lebiouse, A., Virginia City, Neb.
Delisser, G. P., New York, N. Y.
Delmed, —, V.S., Chicago, Ill.
Demeritt, W., Dover, N. H.
Denslow, I., Brockport, N. Y.
Derby, E. I., West Alexandria, O.
Derby, M. C., Manchester, N. H.
Derr, W. F., V.S., Wooster, O.
De Tavel, —, San Francisco, Cal.
Devlin, C. F., Philadelphia, Pa.
Dillon, E. & Co., Bloomington, Ill.
Ditmer, H. J., D.V.S., Chicago, Ill.
Dixon, D. I., St. Thomas, Pa.
Dobson, W. H., Cincinnatus, N. Y.
Dods, A. W., Silver Creek, N. Y.
Domeek, A., Louisville, Ky.
Doney, H. W., Jackson, Mich.
Doman, D. H., Milwaukee, Wis.
Dorsey, A. & I., Perry, Ill.
Doty & Sladden, Detroit, Mich.
Dougherty, W.,V.S., Baltimore, Md.
Douglas, A. G., Perth, Ont.
Duoglas, —, Brooklyn, N. Y.
Douglas, —, Jersey City, N. J.
Dowling, W., Detroit, Mich.
Dreibelbis, P. K., Lenhardtsville, Pa.
Drinkwater, A., Rochester, N. Y.
Duane, —, New York, N. Y.
Dunne, C., D.V.S., New York, N. Y.
Dyer, C. K., D.V.S., Mount Holly, N. J.
Dysart, S., Franklin Grove, Ill.
Earle, H. E., M.D., V.S., New York, N. Y.

Earnest, J., Columbus, O.
Easton, W., New York, N. Y.
Eck, M., Chicago, Ill.
Eckstein, G. W., Philadelphia, Pa.
Edwards, W. G., New Orleans, La.
Eggleston, W. M., Chicago, Ill.
Elkins, O. H., North Troy, Vt.
Elliott, J., Toledo, O.
Ellis, J. H., Philadelphia, Pa.
Emmett, C. F., New Orleans, La.
Ennever, W. C., New York, N. Y.
Esslement, W., New York, N. Y.
Estelle, J. T., Cincinnati, O.
Evans, G. A., San Francisco, Cal.
Evans, L. D., Munson, Ind.
Fair, W. C., Cleveland, O.
Fairchild, D. S., Ames, Iowa.
Faivre, C., Philadelphia, Pa.
Falen, T., Chicago, Ill.
Faley, M., Philadelphia, Pa.
Farley, O. C., D.V.S., Boston, Mass.
Farness, O. O., Ortonville, Minn.
Farrell, S., St. Louis, Mo.
Farwell, I. V., Lake Forest, Ill.
Fauria, —, Pensacola, Fla.
Fausse, A., Montreal, Can.
Fawns, C., Newark, N. J.
Fay, F. V., V.S., Millbrook, O.
Fenner, H., Newark, N. J.
Ferdinandson, J., Newark, N. J.
Ferrell, H. Y., Savannah, Ga.
Ferries, J., D.V.S., Beaverly, Ont.
Field, —, Chicago, Ill.
Field, H., Fall River, Mass.
Field, S. S., D.V.S., New York, N. Y.
Fife, C. C., Boston, Mass.
Finlay, —, New York, N. Y.
Finlay, R. W., New York, N. Y.
Finn, B., New York, N. Y.
Fitzgerald, E., D.V.S., New York, N. Y.
Flagg, O. H., New Bedford, Mass.
Flintoff, H. I., New York, N. Y.
Fly, J. C., Philadelphia, Pa.
Fogg, J. C., D.V.S., Boston, Mass.
Foote, H. F., New York, N. Y.
Foote, J. H., New York, N. Y.
Force, J. C., D.V.S., Newark, N. J.
Fordham, E., Brooklyn, N. Y.
Foreacre, M., Bolivar, O.
Frank, M., Baltimore, Md.
Frazer, —, New York, N. Y.
French, L., Williamson, N. Y.
French, W., New Orleans, La.
Frey, M. L., D.V.S., New York, N. Y.
Frey, R., St. Paul, Minn.
Frink, D. D., V.S., St. John, N. B.
Frink, J. H., V.S., St. John, N. B.
Frisom, O., Omaha, Neb.
Fuller & McIlroy, Grand Rapids, Mich.
Fuller, R. C., Dayton, O.
Gadsden, J. W., M.R.C.V.S., Philadelphia Pa.
Gaffley, A., Kansas City, Mo.
Gaines, J., Saco, Me.
Galt, J. B., Morrison, Ill.
Gantz, P., Chicago, Ill.
Garity, P., Chicago, Ill.
Garman, J. G., V.S., Union Station, Pa.
Gaulding, F. L., Port Huron, Mich.
Gerety, M., St. Louis, Mo.
Gerth, J., Jr., Newark, N. J.
Gibbs, W., St. Mary's, Ont.
Gibson, G., Pittsburg, Pa.
Gifford, C., New York, N. Y.
Gillett, I. M., Jr., Spencer, Ill.
Girard, F., Philadelphia, Pa.
Glasgow, T., Philadelphia, Pa.
Goad, H. A., Columbus, O.
Going, J. A., M.R.C.V.S., New York, N. Y.
Going, J. B., Fort Clark, Texas.
Gosling, J., Columbus, O.
Gottleib, A. T. E., Utica, N. Y.
Gove, J. C., Hampton Falls, N. H.
Greenside, F. C., V.S., Toronto, Can.
Gregson, J., St. Louis, Mo.
Greiner, L. A., Indianapolis, Ind.
Grice, J. C., New York, N. Y.
Grun, J. B., Montreal, Can.
Guenther, F., St. Louis, Mo.
Guesinger, A., Chicago, Ill.
Haas, J., Dayton, O.
Haas, J., Indianapolis, Ind.
Haensler, J., Buffalo, N. Y.
Hall, C. H., D.V.S., New York, N. Y.
Hall, C. P., New York, N. Y.
Hall, C. P., Swanton, O.
Hall, R., New York.
Hall, R. C., Webster, Mass.
Hall, R. W., D.V.S., Fanwood, N. J.
Hall, W. B., Quebec, Can.
Hamill, J. D., D.V.S., New York, N. Y.
Hamlin, J., Wilkesbarre, Pa.
Hanshew, E. J., Jr., D.V.S., Brooklyn, N. Y.

Harding, J., Hastings, Neb.
Harrison, R., D.V.S., New York, N.Y.
Harthill Bros., Louisville, Ky.
Harvey, G., Pittsburg, Pa.
Harvey, G. W., Boston, Mass.
Hastings, S. B., West Finley, Pa.
Hawk, J. W., Newark, N. J.
Hawkins, J., Detroit, Mich.
Haydon, W. B., Newark, N. J.
Heard, E. H., Brooklyn, N. Y.
Heard, J., Cleveland, O.
Heard, J. M., M.R.C.V.S., New York, N. Y.
Herbert, S., D.V.S., St. John, P. Q.
Heischman, ——, Gahanna, O.
Herser, E., Pottsville, Pa.
Herst, W. C., V.S., Ashtabula, O.
Hempelman, H., St. Louis, Mo.
Henderson, A. & Son, Syracuse, N.Y.
Hennings, C., Fremont, Neb.
Herand, J. L., Logansport, Ind.
Herr, L., Lexington, Ky.
Herr, T. J., Brooklyn, N. Y.
Herr, T. J., D.V.S., New York, N.Y.
Hesse, C. H., St. Louis, Mo.
Hill, C. J., Arlington, Md.
Hindekoper, R. S., Philadelphia, Pa.
Hinkley, N. P., D.V.S., Buffalo, N.Y.
Hison, W. C., V.S., Dixon, Ill.
Hetchman, W. H., Philadelphia, Pa.
Hodgdon, C. A., Lancaster, N. H.
Hodgson, T., Toronto, Can.
Hoeing, C. F., Jersey City, N. J.
Hoffman, J. J., Philadelphia, Pa.
Hoglen Bros., Dayton, O.
Holbert, A., Cleveland, O.
Holcombe, A. A., D.V.S., Fort Leavenworth, Kan.
Holladay, H. M., Detroit, Mich.
Hollman, ——, Brooklyn, N. Y.
Holpape, ——, Hoboken, N. J.
Holzschurch, H. H., New York, N.Y.
Homberg, J., New York, N. Y.
Home, W., M.D. V.S., Janesville, Wis.
Hopkins, A. J., Hartford, Ct.
Hopkins, J. D., D.V.S., New York, N. Y.
Hornblower, W., New York, N. Y.
Hornblower, W. H., D.V.S., Arlington, Mass.
Hoss, C., St. Louis, Mo.
Hostetter, C. L., Mount Carroll, Ill.
Houghton, G. S., New York, N. Y.
Houghton, L., Spencerville, Ind.
House, C. D., D.V.S., New York, N.Y.
Houseman, H., V.S., Brooklyn, N.Y.
Howe, ——, V.S., New York, N. Y.
Howell, G. J., Desmoines, Iowa.
Hull, F. M., Chicago, Ill.
Hull, G. G., Newport, R. I.
Hunt, G. W., Greenwood, Ill.
Huntsberger, W., East Union, O.
Hurbutt, N., Lebanon, N. H.
Hutchins, R. C., Buffalo, N. Y.
Hutchinson, W. W., Racine, Wis.
Hyland, J., Baltimore, Md.
Ifield, T., Washington, D. C.
Imbourg, C., San Francisco, Cal.
Imbouy, C., V.S., New York, N. Y.
Jackson, O. C., D.V.S., Jamaica, L. I., N. Y.
Jacobson, I., San Francisco, Cal.
Jacobus, T. O., Newark, N. J.
Jakeman, W., D.V.S., Halifax, N.S.
James, D., Philadelphia, Pa.
Jennings, J. H., Cambridgeport, Mass.
Jennings, R., Detroit, Mich.
Jennings, R., Pittsburg, Pa.
Jerome, ——, Saginaw, Mich.
Jeter, J. S., Yanceyville, N. C.
Johnson, E. L., Pennington, N. J.
Johnson, J. W., Cleveland, O.
Johnson, J. W., V.S., Phila., Pa.
Jones, I., St. Paul, Minn.
Jones, W. A., Buffalo, N. Y.
Joynes, A., Weldon, N. C.
Karrigan, J., Brooklyn, N. Y.
Keller, J. J., St. Louis, Mo.
Kelly, W., Utica, N. Y.
Kendall, B. I., Enosburg Falls, Vt.
Kendrick, T. B., Omaha, Neb.
Kennedy, W. E., Knoxville, Ill.
Kidney, G. H., V.S., Swanton, Vt.
Kilfride, G., Philadelphia, Pa.
Kilmer, J., D.V.S., Winesburg, O.
Kingey, E. H., V.S., Arlington, Ill.
Kleindorf, W. H., D.V.S., Middletown, Pa.
Knoblaugh, J. N., V.S., Geneseo, Ill.
Knott, J., Chicago, Ill.
Kooker, W. S., Philadelphia, Pa.
Kraul, A., V.S., Elmira, N. Y.
Kuenzli, J., New York, N. Y.
Kungle, D. E., V.S., New York, N.Y.
Kuntz, A., New York, N. Y.
Kylander, C. H., Woburn, Mass.
Laidlaw, R., Albany, N. Y.
Laird, G. T., Detroit, Mich.
Lanephere, R., East Saginaw, Mich.

LaPort, J., Cleveland, O.
Large, —, New York, N. Y.
Lauch, E., Newark, N. J.
Law, J., D.V.S., Ithaca, N. Y.
Lawrenz, C., Newark, N. J.
Lehman, J. Y., Sterling, Ill.
Leimer, F. J., Chicago, Ill.
Leis, R., Newark, N. J.
Lejambre, H., Philadelphia, Pa.
Lemas, D., Baltimore, Md.
LeMaster, L., Honey Grove, Texas.
Lemay, D., D.V.S., Baltimore, Md.
Lenhart, C., Dover, Pa.
Levesque, A., D.V.S., Montreal, Can.
Levesque, C., D.V.S., Berthier, Can.
Levi, E. S., D.V. S., Dubuque, Iowa.
Liautard, A., M.D., V.S., New York, N. Y.
Light, D. K., D.V.S., Lebanon, Pa.
Lillyman, W. H., Boston, Mass.
Lindeman, —, Boston, Mass.
Lindsay, J., D.V.S., Huntingdon, L. I., N. Y.
Linthicum, C. G., Baltimore, Md.
Lippincott, T., Burlington, N. J.
Lochbiler, C., St. Louis, Mo.
Lockhart, —, New York, N. Y.
Lord, R. P., Baltimore, Md.
Lowell, G. V., Buffalo, N. Y.
Luce, E. S., Chicago, Ill.
Luffbarry, J. C., Philadelphia, Pa.
Luse, J. P. & Son, Montmorency, Ind.
Lyford, C. C., Minneapolis, Minn.
Lyman, C. P., Springfield, Mass.
Lynch, P., Newark, N. J.
Lyon, L. Z., Baltimore, Md.
Lyons, —, New York, N. Y.
McBain, W. E., Toledo, O.
McCarthy, P., Chicago, Ill.
McClean & Son, Brooklyn, N. Y.
McCoart, J., Philadelphia, Pa.
McCorack, A., D.V.S., Ormiston, P.Q.
McCormack, C. B., Tideoute, Pa.
McDonald, C., New York, N. Y.
McDonnel, J. J., Chicago, Ill.
McDougal, W., Pleasant Hill, Ill.
McEachran, W., M.D., C.M., Montreal, Can.
McFarland, G., Philadelphia, Pa.
McGehee, J., Milton, Pa.
McGregor & Lewis, San Francisco, Cal.
McInnes, B., Charleston, S. C.
McInroy, E. B., Chicago, Ill.
McIntosh, J., Montgomery, Ala.
McKenzie, A., Cleveland, O.
McKenzie, J. C., Rochester, N. Y.
McKillop, M. H., Chicago, Ill.
McLaughlin, J., Buffalo, N. Y.
McLaughlin, J. A., Jr., D.V.S., Jersey City, N. J.
McLaughlin, J. R., D.VS., Lynn, Mass.
McLean, R. A., D.V.S., Brooklyn, N.Y.
McLellan, E. A., V.S., Bridgeport, Ct.
McLellan, F. W., D.V.S., Bridgeport, Ct.
McMartin, H. J., D.V.S., Potsdam, N. Y.
McQuiney, L., Buffalo, N. Y.
McWort, J., Philadelphia, Pa.
Manly, M., D.V.S., Mountainville, N. Y.
Manning, J. B., Rochester, N. Y.
Mansback, L. A., Philadelphia, Pa.
Markley, A. & Son, Dayton, O.
Marsh, A. B., Mount Vernon, N. Y.
Marshall, J. A., Philadelphia, Pa.
Marshall, W., San Francisco, Cal.
Martin, A. S., D.V.S., Cleveland, O.
Martin, D. G., Martinsdale, Pa.
Martin, T. J., Urbana, O.
Martinet, W. H., Baltimore, Md.
Mason, J. C., Annapolis, Ill.
Mather, L. C., Academy, N. Y.
Matterson, M. G., D.V.S., Pettstown, N. J.
Maxwell, E., Portland, Me.
Maynard, H. G., Hartford, Ct.
Mellor, A., Philadelphia, Pa.
Mellor, G., Philadelphia, Pa.
Melms, H., Milwaukee, Wis.
Merrill, D. K., V.S., Harrison, O.
Merry, M., Holyoke, Mass.
Metzenius, O. W., New York, N. Y.
Metzer, S., Chicago, Ill.
Meyer, C. A., D.V.S., New York, N. Y
Meyer, J., Jr., Cincinnati, O.
Meyer, J. C., Cincinnati, O.
Michener, C. B., V.S., New York, N. Y.
Middlebrook, E. B., New York, N.Y.
Miles, I. J., D.V.S., Charleston, Ill
Miles, W. L., Champagne, Ill.
Miller, A., Chicago, Ill.
Miller, B E., Camden, N. J.
Miller, D. R., V S., Nashville, O.
Miller, W. B. E., D V.S., Metuchin, N J.
Milloy, J., D.V.S., Boston, Mass.

Mills, C., New York, N. Y.
Millspaugh, J., St. Louis, Mo.
Milnes, J. C., Cedar Rapids, Ill.
Mink, E., Rochester, N. Y.
Mitchell, A., Baltimore, Md.
Mitchell, L A., Clinton, Ill.
Moody, P. M., Belfast, Me.
Moore, A. B., Jr., Scio, O.
Moore, A. C., Canton, Ill.
Moore, E., M.R.C V.S., Albany, N.Y.
Moore, H., Albany, N Y.
Moore, W. O., M.D.D.V.S., New York, N. Y.
Moran, J., Newark, N. J.
Morse, G. B., Boston, Mass.
Moseley, D. C., Baltimore, Md.
Mosher, G. W., Presque Isle, Me.
Moore, H. C., Troy, N. Y.
Moyan, P. R., Chicago, Ill.
Mudgett, A. H., Minneapolis, Minn.
Muller, E., Grand Island, Neb.
Mulloy, J. C., Chelsea, Mass.
Mulroy, J. C., Chelsea, Mass.
Munro, J. M., San Francisco, Cal.
Murphy, J., Stoughton, Mass.
Murphy, W. A., D.V.S., Cambridge, Mass.
Murray, A. J., V.S., Detroit, Mich.
Murray, P., New York, N. Y.
Mustoe, G. F., D.V.S., Brooklyn, N. Y.
Myers, J. C., Jr., V.S., Cincinnati, O.
Myers, T. S., Baltimore, Md.
Myrman, A. G.. Chicago, Ill.
Nagelfield, J., Detroit. Mich.
Navin, J. N., V.S., Indianapolis, Ind.
Neebe, H., New York, N. Y.
Nell, G., New York, N. Y.
Nevitt, J., Chicago, Ill.
Newton, J. V., V.S., Toledo, O.
Nichols, W. W., Brooklyn, N. Y.
Nidal, R., San Francisco, Cal.
Nixon, E., Chicago, Ill.
Nostrand, E., D.V.S., New York, N. Y.
Nostrand, P., V.S., New York, N.Y.
Noyes, W. W., Boston, Mass.
O'Donnell, J., Chicago, Ill.
Ogle, —, New York, N. Y.
O'Halloran, P., New York, N. Y.
O'Neill, J., San Bernardino, Cal.
Ormond, C. H., D.V.S., Milwaukee, Wis.
Ormond, W. M., Milwaukee, Wis.
Osborn, H., Ames, Iowa.
O'Shea, —, New York, N. Y.
Ottofy, L., St. Louis, Mo.
Overmiller, J. F., Logansville, Pa.
Owen, O. W., Sunol, Cal.
Paaren, N. H., Chicago, Ill.
Packard, A. S., Brookline, Mass.
Page, J., D.V.S., Lotbinière, P. Q.
Palmer, C. E., New Haven, Ct.
Palmer, G. G., New York, N. Y.
Palmer, T. S., Washington, D. C.
Parker, C. H., Minneapolis, Minn.
Parker, G. Troy, N. Y.
Parker, I. H., New York, N. Y.
Parker, L. N., Minneapolis, Minn.
Parker, T., Troy, N. Y.
Parker, W. H., Yates City, Ill.
Parkinson, G. H., D.V.S., Middletown, Ct.
Parshall, S. B., Canfield, O.
Patterson, J. W., Detroit, Mich.
Patterson, W., M.R., C.V.S., Montreal, Can.
Paulson, W. J., D.V.S., Flemington, N. J.
Peabody, C. H., D.V.S., Providence, R. I.
Penniman, G. B., D.V.S., Worcester, Mass.
Perry, M. A., Troy, N. Y.
Perry, S., Troy, N. Y.
Peter, P. S., M.D., Yanceyville, N.C.
Peters, P., V.S., Fort Ringgold, Texas.
Phillips, A. T., Wyandotte, Mich.
Phillips, A. W., Indianapolis, Ind.
Pick, E. J., D.V.S., New York, N.Y.
Picquet. L., McLeansboro, Ill.
Pierce, B. D., D.V.S., Springfield, Mass.
Pitcher, A. D., Glenn's Falls, N. Y.
Plageman, L. V., Brooklyn, N. Y.
Plummer, E., Baltimore, Md.
Pollard, W. H., Burlington, Vt.
Poole, A. S., Long Branch, N. J.
Poole, S., New York, N. Y.
Porter, J. L., Montgomery, Ala.
Postell, R., St. Paul, Minn.
Potter, C. C., Boston, Mass.
Potter, W. A., Providence, R. I.
Powell, W., Beecher, Ill.
Powers, T. H., Brooklyn, N. Y.
Prentiss, G. B., Kansas City, Mo.
Prentiss, T. W., Urbana, Ill.
Prevost, V., D.V.S., Sherbrooke, P.Q.
Price, R., D.V.S., St. Paul, Minn.
Pritchard, E. H., Indianapolis, Ind.

Prive, P., M.D.V.S., Terre Bonne, P. Q.
Pulson, W., Flemington, N. J.
Purdy, G. W., Louisville, Ky.
Putnam, L., North Cambridge, Mass.
Putney, C. M., San Jose, Cal.
Quintin, C., Plainfield, N. J.
Radecke, E. F., St. Louis, Mo.
Raines, E., Clinton, Ill.
Ramsey, S. V., Parkville, Ill.
Ramus, E., V.S., Clinton, Ill.
Randall, I. H., Willard, N. Y.
Rape, M., Cincinnati, O.
Rathborn, C. D., Sherwood, Mich.
Ray, W. H., Dobb's Ferry, N. Y.
Raynor, G. B., Philadelphia, Pa.
Raynor, T. B., Philadelphia, Pa.
Rea, F., Wortendyke, N. J.
Read, H. A., Fort Wayne, Ind.
Redhead, H. W., Cleveland, O.
Redmond, P., Chicago, Ill.
Reed, J. M., Majority Point, Ill.
Reed, W., Jersey City, N. J.
Reigle, J. W., Plain View, Pa.
Reinhart, N. E., Steel Works, Pa.
Reynolds, J. S., Chicago, Ill.
Ribiere, J., Washington, D. C.
Roberge, F. P., D.V.S., New York, N. Y.
Robertson, J. S., M.D., V.S., New York, N. Y.
Robie, W. F., D.V.S., Manchester, N. H.
Robinson, C. B., St. Thomas, Ont.
Robinson, F., Amherstburg, Canada.
Rodfong, G. W., Philadelphia, Pa.
Roe, A. M., Grand Rapids, Mich.
Rogers, G. G., Rochester, N. Y.
Rogers, H. G., Pittsburg, Pa.
Rogers, T. B., D.V.S., Newark, N. J.
Rose, A. H., D.V.S., New Haven, Ct.
Rose, J., Columbus, O.
Rose, W., Tompkinsville, S. I., N.Y.
Rose, W. H., D.V.S., Stapleton, S. I., N. Y.
Rosenstiel, J. I. J., Champagne, Ill.
Rosenthal, H. A., Brooklyn, N. Y.
Ross, L. F., Avon, Ill.
Rothbarth, P., Chicago, Ill.
Rowland, E. W., Miller's Place, L. I., N. Y.
Roy, L. G., Front Royal, Va.
Ruktenwaldt, N., V.S., Pittsburg, Pa.
Russell, R. W., Kearney, Neb.
Ryan, J. F., D.V.S., Chicago, Ill.
Salisbury, G. L., V.S., Providence, R. I.
Sallade, I. W., Reading, Pa.
Salmon, D. E., D.V.S., Swannanoa, N. C.
Sanborn, A. H., Chicago, Ill.
Sappel, J., Philadelphia, Pa.
Saunders, J. S., Boston, Mass.
Saunders R. J., Salem, Mass.
Saunders, W., Salem, Mass.
Saunders, W., Boston, Mass.
Saunders, W. E. J., Jersey City, N. J.
Savidan, J., San Jose, Cal.
Schaefer, P., Baltimore, Md.
Schaffner, J. J., Chicago, Ill.
Scheele, E. H., St. Louis, Mo.
Scheele, L., St. Louis, Mo.
Scheffer, D. M., V.S., Nettle Creek, Ind.
Schlattman, J. A., St. Louis, Mo.
Schmidt, G., Newark, N. J.
Schmidt, W. G., Newark, N. J.
Schoeneman, F., Milwaukee, Wis.
Schroeder, T., Chicago, Ill.
Schueler, C., Milwaukee, Wis.
Schulhofer, G., Richmond, Va.
Schultz, M., Brickerville, Pa.
Scully, G., St. Louis, Mo.
Seavy, A. J., Dover, N. H.
Senti, J., Milwaukee, Wis.
Sermon, G., Minneapolis, Minn.
Sessions, N., Worcester, Mass.
Sewell, E. N., D.V.S., New York, N. Y.
Sexton, P., St. Louis, Mo.
Sharpnaed, W. H., Carmichaels, Pa.
Shauler & Bowman, Detroit, Mich.
Shipman, F. B., V.S., Wilmington, O.
Sheridan, G. B., St. Louis, Mo.
Sherlock, E. T., Sutton, Neb.
Siegeler, C., Brooklyn, N. Y.
Sieverling, J. W., Omaha, Neb.
Siler, —, Brooklyn, N. Y.
Simmons, S., Boston, Mass.
Simmons, S., Lynn., Mass.
Simons, T., V.S., New York, N. Y.
Simpson, W. R., St. Louis, Mo.
Skaggs, A. E., Townsend, Del.
Skally, J., Montreal, Can.
Sloan, A., St. Louis, Mo.
Smith, A. (Prof.), Toronto, Can.
Smith, F. S., Fort McIntosh, Texas.
Smith, H. P., Edwardsville, Ill.
Smith, I., New York, N. Y.
Smith, J. J., D.V.S., Chamb'sb'g, Pa.

Smith, J. J., V.S., Portland, Oregon.
Smith, L., Deerlick, O.
Smith, R., Syracuse, N. Y.
Smith, R. & Co., Washington, D. C.
Smith, W. T., Middlebury, Vt.
Somerville, S., Buffalo, N. Y.
Somerville, W. & Sons, Buffalo, N.Y.
Somerville, W., Jr., Buffalo, N. Y.
Sould, T., St. Paul, Minn.
Spankling, C. W., Baltimore, Md.
Sparkhall, R. H., Detroit, Mich.
Spencer, C. A., Dallas, Pa.
Spencer, M. L., Little Geneseo, N.Y.
Sprankling, C. W., Louisville, Ky.
Stack, J., Syracuse, N. Y.
Staffeo, F., M.R.C.V.S., Hamilton, Ont.
Stafford, R., Nebraska City, Neb.
Stalker, M., Ames, Iowa.
Steck, C. A., Jr., Baltimore, Md.
Stein, C. A., St. Paul, Minn.
Steuber, C., Jersey City, N. J.
Stevens, E. H., Syracuse, N. Y.
Stickney, J. H., Boston, Mass.
Stocker, C. V., V.S., Salem, Mass.
Stokes, ——, New York, N. Y.
Stratton, S., Litchfield, Ill.
Strutton, Providence, R. I.
Stryck, E. O., New York, N. Y.
Stuart, E. P., Naples, N. Y.
Stuart, G., Cleveland, O.
Stuart, L. W., Monmouth, Iowa.
Stutterheim, J., Philadelphia, Pa.
Sudlow, S., Brooklyn, N. Y.
Sutherland, D. G., Franklin, Mich.
Swan, ——, New York, N. Y.
Sybertz, J., Belleville, Ill.
Taede, W., Phillipsburg, N. J.
Talbot, B., New York, N. Y.
Taylor, C. C., Milwaukee, Wis.
Taylor, J. E., Toledo, O.
Taylor, J. N., Utica, N. Y.
Taylor, T., Washington, D. C.
Tegg, A., Rochester, N. Y.
Tegtmeier, A., Philadelphia, Pa.
Thayer, ——, V.S., West Newton, Mass.
Thom, J., Baltimore, Md.
Thomas, F. S., M.D.V.S., Hanson, Mass.
Thomas, W., Trenton, Tenn.
Thompson, G. P., Baltimore, Md.
Thompson, J. F., Canton, Ill.
Thompson, N. F., D.V.S., New York, N. Y.
Thorne, J., Baltimore, Md.
Threipland, J., Chicago, Ill.
Throup, R. D., Cleveland, O.
Thurman, G. H., St. Louis, Mo.
Timberman, J. H., Philadelphia, Pa.
Titus, H. B., Pawtucket, R. I.
Tjaden, C., David City, Neb.
Tolkaez, M., St. Louis, Mo.
Tolleo, H. W., Nashua, N. H.
Townsend, N. S., M.D., Columbus, O.
Traver, E., V.S., Jobstown, N. Y.
Travis, F., Rhinebeck, N. Y.
Trudel, N. A., D.V.S., Three Rivers, P. Q.
Trumbower, M. R., Sterling, Vt.
Turner, J., Toronto, Can.
Tuthill, J. D., Chicago, Ill.
Twohig, J., Fort Verde, Arizona.
Tyford, C. C., Minneapolis, Minn.
Van Arnum, M., V.S., Wright's Corners, N. Y.
Vance, G. A., Westerville, O.
Vandenhoff, H. S., M.D., D.V.S., Williamsburg, N. Y.
Vandercook, W. C., Cherry Valley, N. Y.
Van Sittert, —, Desmoines, Iowa.
Vasey, N., Troy, N. Y.
Vaughn, S. W., Richmond, Va.
Verleger, C. F., Baltimore, Md.
Very, T. S., Boston, Mass.
Viles, J. A., Lowell, Mass.
Virtue, S., V.S., Iberia, O.
Vogel, A., Chicago, Ill.
Vogeler, C. D., Newark, N. J.
Vohden, S., Baltimore, Md.
Volz, C., Newark, N. J.
Von Krause, A., Stratford, Ct.
Waddell & Benedict, Columbus, O.
Waldbart, J., Milwaukee, Wis.
Walker, M. R., Newark, N. J.
Wallace, J. C., D.V.S., New York, N. Y.
Wallenborn, P. A., Chicago, Ill.
Walling, J. Z., Cincinnati, O.
Wallis, E., Rochester, N. Y.
Walther, F. H., Boston, Mass.
Walton, F., D.V.S., New York, N.Y.
Warburton, A., Franklin Falls, N.H.
Wasteny, J. G., Cincinnati, O.
Waterhouse, J., Stockton, Cal.
Waters, E., Brooklyn, N. Y.
Watson, H. P., M.D., North Haverhill, N. H.
Watson, W., Philadelphia, Pa.

Weddell, R. W., Aurora, Ill.
Weeks, A. P., D.V.S., Ellenville, N. Y.
Weidner, J. N., Baltimore, Md.
West, J., St. Louis, Mo.
Wettleson, Wm. O., V.S., Storighton, Dane Co., Wis
Wheat, L. E., V.S., Scranton, Pa.
White, G. B., Providence, R. I.
White, H. A., New Orleans, La.
White, M., V.S., Weston, O.
White, S. H., Cleveland, O.
Whitehead, E., Cincinnati, O.
Whitehouse, J., Rochester, N. Y.
Whitney, W. J., Brayton, Iowa.
Whitney, W. S., New Haven, Ct.
Whitridge, G. R., Mt. Pleasant, S. C.
Whittlesey, R. T., Emporia, Kansas.
Wilber, N. H., M.D., Troupsburg, N. Y.
Wilbur, C., Providence, R. I.
Wilbur, G. F., M.D., Hightstown, N. J.
Wilcox, C. O., Chicago, Ill.
Wiley, W., Andersontown, Pa.
Wilkinson, J. B., M.D., Wood Park, La.
Wilkinson, F. C., Claremont, N. H.
Williams, H., Racine, Wis.
Williams, H., San Francisco, Cal.
Williams, H. M., Mapleton, Dakota.
Williams, T., Washington, D. C.
Williams, W. A., Bloomington, Ill.
Williams, W. L., D.V.S., Bloomington, Ill.
Wilson, F. J., D.V.S., Brooklyn, N. Y.
Wilson, J. A., Tremont, O.
Wilson, W., D.V.S., Jersey City, N.J.
Wilton, A. B., Boston, Mass.
Wilton, G., Boston, Mass.
Winchester, J. F., B.S., Lawrence, Mass.
Wing, E. R., D.V.S., Needham, Mass.
Winnsch, E., Cincinnati, O.
Winslow Bros., Kankakee, Ill.
Winslow, C., D.V.S., Rockland, Mass.
Wirtz, J., Indianapolis, Ind.
Wisdom, R. H. B., Philadelphia, Pa.
Withers, R. J., Chicago, Ill.
Withig, E., Jersey City, N. J.
Witleycombe, J., Hillsboro, Oregon.
Wittig, ——, Brooklyn, N. Y.
Wood Bros., Chicago, Ill.
Wood, C. R., Manchester, N. H.
Wood, J., Newburg, N. Y.
Wood, R., V.S., Lowell, Mass.
Woodruff, J. E., Washington, D. C.
Woods, B., V.S., Sherman, Mich.
Work, F. T., Cleveland, O.
Work, J. M. & S., Bardolph, Ill.
Wray, W. H., D.V.S., Yonkers, N.Y.
Wright, A., San Francisco, Cal.
Wyslie, T., Philadelphia, Pa.
Yallop, J., Milwaukee, Wis.
Yerden, M., Cleveland, O.
York, A., V.S., Detroit, Mich.
Youkman, J., Cleveland, O.
Young, J. S., Chicago, Ill.

